# Regulatory challenges and current policy for cardiotoxicity detection in oncology drug development

**DOI:** 10.1007/s11239-026-03337-w

**Published:** 2026-05-29

**Authors:** Richard C. Becker

**Affiliations:** University of Cincinnati College of Medicine, 231 Albert Sabin Way, Cincinnati, OH 45267 USA

**Keywords:** Cancer Therapeutics, Regulatory Agencies, Pharmacovigilance, Cardiotoxicity

## Abstract

The rapid evolution of oncology therapeutics, particularly targeted agents, antibody–drug conjugates, and immunotherapies has outpaced existing regulatory frameworks for detecting and managing cardiotoxicity. Current gaps include inconsistent cardiac safety requirements across development phases, limited incorporation of Cardio-Oncology endpoints within expedited approval pathways, and inadequate harmonization of post-marketing signal detection. These deficiencies underscore the need for a modernized, system-level approach that integrates regulatory science with real-world cardiovascular risk. This review examines approval processes of the United States Food and Drug Administration (FDA) and the European Medicines Agency (EMA), including expedited pathways such as Breakthrough Therapy, Accelerated Approval, PRIority Medicines (PRIME), and upstream activities in pivotal trials. It highlights their strengths in pre-approval drug dose safety and reducing time-to-therapy while critically evaluating limitations in pre-approval cardiac phenotyping, reliance on surrogate endpoints for cardiotoxicity assessment, and constrained post-market surveillance infrastructures that delay informed, patient-centered understanding of safety and efficacy following drug approval.

## Introduction

The development and approval of cancer therapeutics follow a structured, multi-phase process encompassing preclinical evaluation, clinical trials, and post-marketing surveillance. Regulatory agencies such as the United States (U.S.) Food and Drug Administration (FDA) and the European Medicines Agency (EMA) employ rigorous frameworks to assess safety and efficacy, supported by pharmacovigilance systems including FDA Adverse Event Reporting System.

The FDA adverse event drug reporting system (FAERS), European Union’s pharmacovigilance system (EudraVigilance), and World Health Organization’s global database (VigiBase) [1]. Despite these safeguards, cardiotoxicity remains under-recognized during early development and approval stages. Contributing factors include reliance on surrogate endpoints, exclusion of high cardiovascular-risk patients from early phase and pivotal trials, and limited integration of cardiovascular safety measures in expedited pathways such as Accelerated Approval, Breakthrough Therapy, and PRIority Medicines (PRIME)- an initiative by the EMA designed to provide enhanced support for the development of medicines that target unmet medical needs [2].

While current processes accelerate access to life-saving therapies, they often lack systematic cardiac phenotyping and long-term follow-up, leading to delayed recognition of cardiovascular risks. Post-marketing surveillance systems and real-world evidence have proven essential for identifying late or rare toxicities, yet these mechanisms are inherently reactive and constrained by underreporting and data heterogeneity. Accordingly, proactive strategies are needed, embedding Cardio-Oncology endpoints in trial design, leveraging advanced imaging and biomarkers, and harmonizing global safety standards to ensure that therapeutic innovation aligns with optimal patient safety and efficacy.

This second paper in a three-part series is dedicated to detailing current regulatory processes as a foundation for the final paper, which will propose several innovative strategies and policy reforms designed not only to accelerate drug development but also to better characterize early cardiotoxicity signals and optimize therapeutic balance for informed decision-making by clinicians, regulators, guideline writing groups, and patients.

## Current regulatory framework for oncology drug development

**Fig. 1:**
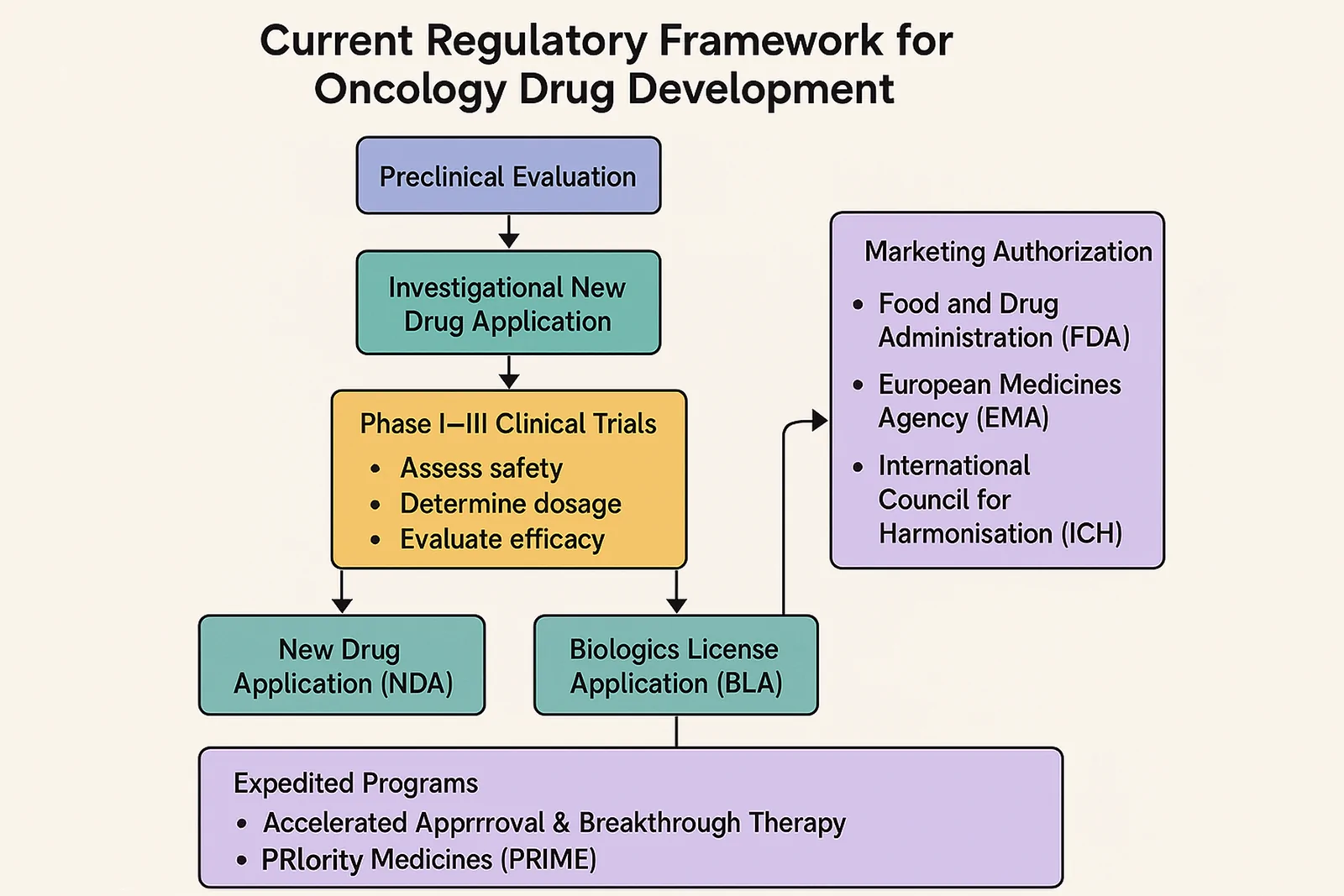
The regulatory process for oncology drug development begins with preclinical studies to establish foundational safety and biological activity, followed by submission of an Investigational New Drug application to enable human trials. These trials progress through three phases, moving from initial safety and dosage assessments to large-scale evaluations of efficacy and adverse effects. Successful completion leads to submission of either a New Drug Application or Biologics License Application for formal review and marketing authorization by agencies such as the FDA and EMA, which operate under harmonized international guidelines. To address urgent patient needs, both agencies offer expedited pathways that accelerate approval for therapies targeting serious conditions with limited treatment options

The approval of cancer therapeutics is governed by structured, multi-phase regulatory pathways designed to ensure safety, efficacy, and manufacturing quality. In the United States, the Food and Drug Administration (FDA) oversees this process through sequential steps beginning with preclinical evaluation, followed by submission of an Investigational New Drug (IND) application, and progression through Phase I–III clinical trials. These trials assess safety, dosage, and efficacy before culminating in a New Drug Application (NDA) or Biologics License Application (BLA). Similarly, the European Medicines Agency (EMA) employs a centralized procedure for marketing authorization, supported by harmonized International Council for Harmonisation (ICH) guidelines. Both agencies utilize expedited programs—such as FDA’s Accelerated Approval and Breakthrough Therapy designations and EMA’s PRIority Medicines (PRIME)—to shorten time-to-therapy for drugs addressing high unmet medical needs (Fig. 1).

While these frameworks have succeeded in accelerating access to life-saving treatments, they exhibit notable limitations in characterizing cardiovascular risk. Current processes often rely on surrogate endpoints, exclude patients with pre-existing cardiac conditions from early phase studies, and lack systematic integration of Cardio-Oncology endpoints in trial design. Post-marketing pharmacovigilance systems, including FAERS, EudraVigilance, and VigiBase, provide essential safety monitoring but remain reactive, identifying cardiotoxicity only after widespread clinical use. These gaps underscore an opportunity to embed cardiovascular safety assessments earlier in development, harmonize global standards, and leverage real-world evidence to inform benefit-risk decisions. Strengthening these processes is critical to achieving a therapeutic balance that optimizes both oncologic efficacy and cardiovascular safety.

### Complementary upstream drug safety activities

The FDA’s *Project Optimus* is an initiative launched by the Oncology Center of Excellence in 2021 aimed at overhauling traditional dose-finding approaches in cancer drug development. Rather than relying on the maximum tolerated dose (MTD), the program mandates early, randomized dose-ranging studies integrating pharmacokinetic/pharmacodynamic data, biomarkers, toxicity (including patient-reported outcomes), and early efficacy signals [3]. Its goal is to identify doses that maximize therapeutic benefit while minimizing harm, emphasizing a scientific, patient-centered dose-selection process well before pivotal trials (www.fda.gov/about-fda/oncology-center-excellence/project-optimus; accessed January 4, 2026).

Since its announcement, *Project Optimus* has proven impactful: uptake of formal dose-optimization plans in early-phase oncology protocols surged from under 20% in 2021 to a majority by 2024, driven by the FDA’s finalized guidance in August 2024. This reflects tangible adoption of its principles, shaping trial designs across industry. As the FDA continues under the current administration to promote evidence-based, patient-focused regulatory frameworks, Project Optimus remains well-positioned to advance its goals [4].

The EMA utilizes a similar process to optimize dose selection for cancer therapeutics [5].

## The key steps in drug development

**Fig. 2:**
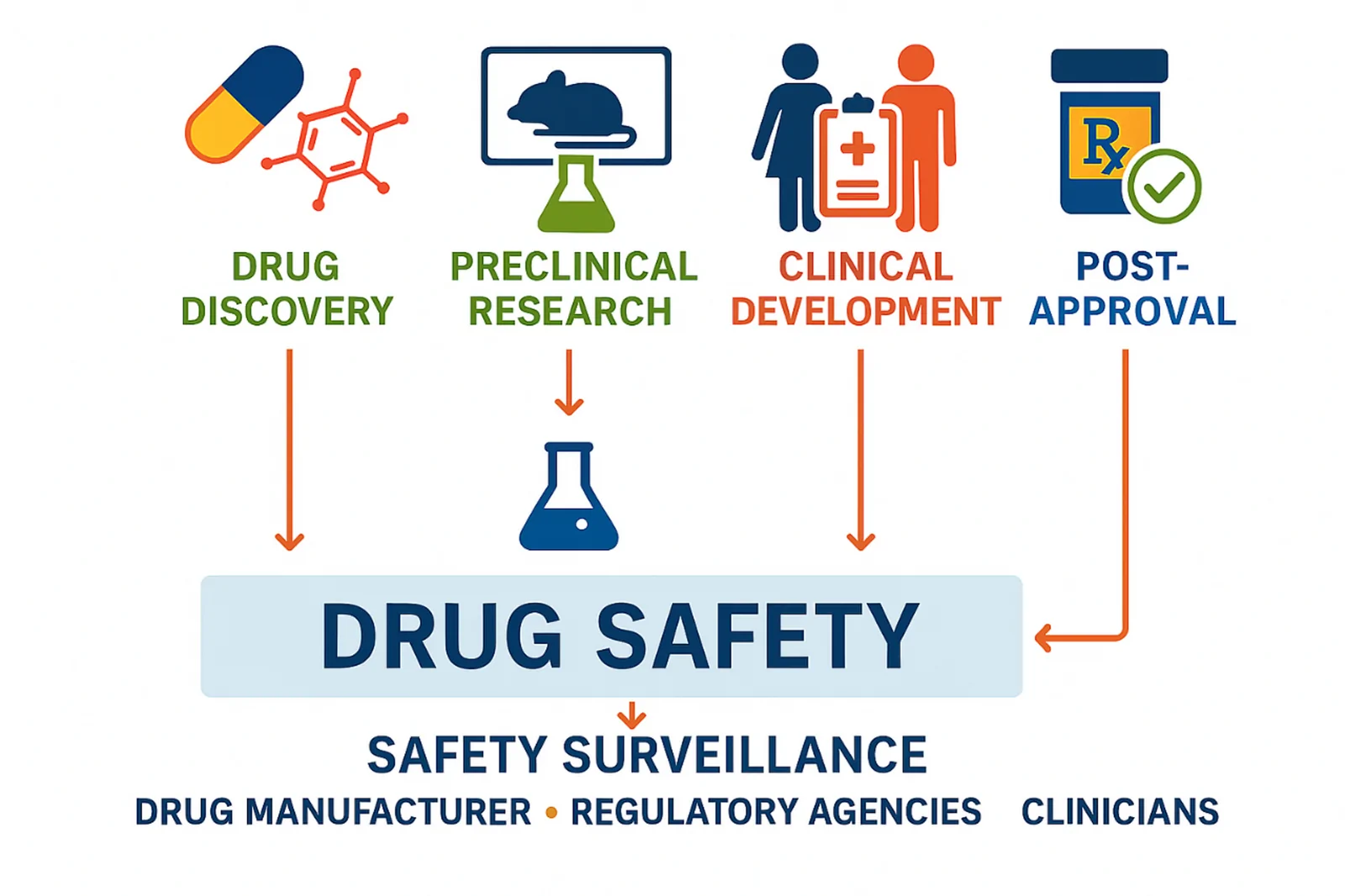
The figure illustrates the drug development lifecycle from initial discovery through post-approval, highlighting the central role of drug safety and the complementary responsibilities of manufacturers, regulatory agencies, and clinicians. The process begins with discovery and preclinical research, where candidate molecules are identified and early safety assessments are conducted. It then progresses to clinical development, encompassing Phase I–III trials that evaluate efficacy and safety under regulatory oversight. Following this, regulatory review and approval focus on benefit-risk evaluation based on manufacturer submissions and clinician input. Finally, post-approval surveillance ensures ongoing pharmacovigilance through real-world evidence collection and collaborative efforts among all stakeholders to monitor and mitigate safety risks

The FDA drug approval process for cancer therapies is a multi-phase, highly regulated pathway that begins with preclinical research and culminates in a Biologics License Application (BLA) or New Drug Application (NDA) (Fig. 2). Once sufficient data is gathered, sponsors submit an Investigational New Drug (IND) application, which must include detailed chemistry, manufacturing, and controls (CMC) information. This covers drug substance characterization, stability data, fill-finish (sterile filling and sealing) processes, and manufacturing protocols. The IND also includes toxicology, pharmacology, and clinical trial protocols. The FDA has 30 days to review the IND and may place a clinical hold if safety concerns arise (www.fda.gov/drugs/investigational-new-drug-ind-application/ind-applications-clinical-investigations-chemistry-manufacturing-and-control-cmc-information; accessed March 14, 2026).

Once the IND is active, the drug enters clinical trials: Phase I (safety and dosage), Phase II (efficacy and side effects), and Phase III (confirmation in larger populations). These trials are overseen by FDA review teams composed of medical officers, toxicologists, statisticians, and chemists who assess safety, efficacy, and manufacturing quality (www.clinicalstudies.in/fda-ind-submission-process-a-complete-sponsor-guide-for-u-s-clinical-trials; accessed January 4, 2026). After successful trials, the sponsor submits a BLA or NDA, which includes all clinical data, labeling, and updated CMC documentation. The FDA reviews the submission, often involving advisory committees, and may grant approval, request more data, or issue a Complete Response Letter. Post-approval, the drug enters Phase IV surveillance to monitor long-term safety (cancerscience.net/knowledgebase/what-is-the-fda-approval-process-for-cancer-drugs; accessed March 14, 2026).

## Cancer therapeutics: Safety assessment

The FDA’s FAERS system is a database maintained by the FDA to support post-marketing safety surveillance for drugs and therapeutic biologics. It contains reports of adverse events and medication errors submitted by manufacturers, healthcare professionals, and consumers. These reports help the FDA monitor drug safety, identify potential safety concerns, and take regulatory actions when necessary (www.fda.gov/drugs/drug-approvals-and-databases/fda-adverse-event-reporting-system-faers-database; accessed March 14, 2026).

EudraVigilance is the European Union’s system for managing and analyzing information on suspected adverse reactions to medicines authorized or being studied in clinical trials within the European Economic Area. It is operated by the EMA and includes two main modules: one for clinical trial data and another for post-authorization safety data. Reports are submitted by pharmaceutical companies, healthcare professionals, and patients. EudraVigilance plays a key role in early detection of safety signals and supports regulatory decisions in the EU (www.ema.europa.eu/en/human-regulatory-overview/research-development/pharmacovigilance-research-development/eudravigilance; accessed March 14, 2026).

Toxicity, in the context of cancer therapeutics, is generally defined by the FDA and the EMA as the degree to which a substance or therapeutic agent can cause harmful effects in patients, encompassing both the nature and severity of adverse events associated with drug exposure [6]-summarized in part 1 of this 3-part series.

The FDA, through its Center for Drug Evaluation and Research (CDER), evaluates toxicity as part of an integrated risk assessment, considering both preclinical (animal, ex vivo) and clinical (human) data [7]. Toxicity is characterized by the occurrence of adverse events, their frequency, severity (graded using standardized criteria such as the Common Terminology Criteria for Adverse Events, CTCAE), reversibility, and impact on organ systems [8]. The FDA does *not* perform quantitative risk extrapolations for pharmaceuticals as is done for environmental chemicals; instead, toxicity is assessed in the context of anticipated clinical benefit, with particular attention to the mode of action and thresholds for toxic effects [9].

The EMA similarly defines toxicity as the spectrum of harmful effects observed in preclinical and clinical studies, with a focus on the safety profile of the drug, including both acute and chronic adverse events. The EMA requires comprehensive safety data, including the incidence, severity, and duration of toxicities, and emphasizes the need for ongoing pharmacovigilance post-approval. The EMA’s approach is harmonized with the FDA through the International Council for Harmonisation (ICH) guidelines, particularly ICH S9, which standardizes nonclinical safety requirements for anticancer pharmaceuticals [10].

Both agencies recognize that the definition and assessment of toxicity must be contextualized within the risk-benefit balance for cancer patients, where higher toxicity may be acceptable if offset by substantial clinical benefit. The incidence of drug toxicity varies by organ system [11] (Table 1).

**Table 1:** Cancer Drug Toxicity by Organ System

Organ System	Therapy Class	Associated Phenotypes (Examples)	Incidence (Any Grade)	Grade 3–5 Toxicity Frequency
Hematologic	Cytotoxic chemotherapy	Neutropenia, anemia, thrombocytopenia	44–69% (serious AE)	10–21% (neutropenia, anemia, thrombocytopenia)
Hematologic	Targeted therapy (TKI)	Cytopenias, anemia, thrombocytopenia	8–15% (serious AE)	8–9% (neutropenia, thrombocytopenia, anemia)
Hematologic	Immune checkpoint inhibitors	Cytopenias, anemia	5–10%	5% (anemia)
Hematologic	CAR-T cell therapy	Cytopenias, neutropenia, anemia	10–15%	10–10% (neutropenia, anemia)
Gastrointestinal	Cytotoxic chemotherapy	Nausea, vomiting, diarrhea, mucositis	30–60%	3–10% (diarrhea, mucositis)
Gastrointestinal	Targeted therapy (TKI)	Diarrhea, hepatotoxicity	10–30%	5–13% (AST/ALT elevation, diarrhea)
Gastrointestinal	Immune checkpoint inhibitors	Colitis, hepatitis	5–15%	5–10% (colitis, hepatitis)
Gastrointestinal	Cytokine therapy	Nausea, vomiting	3%	3% (nausea/vomiting)
**Cardiac**	**Anthracyclines**,** alkylators**	**LV dysfunction**,** arrhythmia**,** heart failure**	**5–10%**	**1–5% (cardiomyopathy**,** arrhythmia)**
**Cardiac**	**HER2-targeted agents**	**LV dysfunction**	**2–7%**	**2–4% (LV dysfunction)**
**Cardiac**	**TKIs (VEGF**,** multi-targeted)**	**Hypertension**,** ischemia**,** heart failure**	**5–15%**	**5–10% (hypertension**,** heart failure)**
**Cardiac**	**Immune checkpoint inhibitors**	**Myocarditis**,** arrhythmia**	**< 1%**	**< 1% (myocarditis)**
Skin	Cytotoxic chemotherapy	Alopecia, rash	20–40%	5–10% (severe rash)
Skin	Targeted therapy (EGFR, BRAF)	Rash, hand-foot syndrome	20–50%	10–20% (severe rash)
Skin	Immune checkpoint inhibitors	Rash, pruritus	10–20%	5–10% (severe rash)
Renal	Platinum agents, TKIs	AKI, proteinuria	5–15%	2–5% (AKI, proteinuria)
Pulmonary	Cytotoxic chemotherapy	Pneumonitis, fibrosis	1–5%	1–2% (pneumonitis)
Pulmonary	Immune checkpoint inhibitors	Pneumonitis	1–5%	1–2% (pneumonitis)
Neurologic	Cytotoxic chemotherapy	Peripheral neuropathy, encephalopathy	10–30%	5–10% (neuropathy)
Neurologic	Immune checkpoint inhibitors	Encephalitis, neuropathy	< 5%	< 1% (encephalitis)

AE: Adverse Event, AKI: Acute Kidney Injury, ALT: Alanine Aminotransferase, AST: Aspartate Aminotransferase, BRAF: B-Rapidly Accelerated Fibrosarcoma (oncogene), CAR-T: Chimeric Antigen Receptor T-cell, EGFR: Epidermal Growth Factor Receptor, HER2: Human Epidermal Growth Factor Receptor 2, LV: Left Ventricular, TKI: Tyrosine Kinase Inhibitor, VEGF: Vascular Endothelial Growth Factor

## The world health organization safety database

VigiBase serves as the World Health Organization’s (WHO) global database for individual case safety reports (ICSRs), collecting adverse drug reaction (ADR) data from over 150 countries and encompassing all organ systems and drug classes [12]. It enables the identification, characterization, and signal detection of cardiac and non-cardiac ADRs through disproportionality analyses and data mining techniques. These include cardiotoxicity, hepatic injury, neuropsychiatric events, dermatologic reactions, hematologic toxicity, and fatal ADRs, among others [13, 14].

The database supports subgroup analyses by age, sex, geography, and other risk factors, allowing for the identification of vulnerable populations and region-specific ADR patterns [15]. VigiBase is instrumental in characterizing fatal ADRs globally [16].

## Regulatory agency roles, responsibilities, and assessment tools

Within the FDA, CDER is the primary division responsible for drug safety, regulatory review, and ongoing surveillance of prescription drugs. CDER oversees the approval process, post-marketing safety monitoring, and communication of safety issues to the public and healthcare professionals. The Office of Drug Safety (now known as the Office of Surveillance and Epidemiology within CDER) specifically manages post-marketing safety surveillance, adverse event reporting, and risk management. The Office of New Drugs is responsible for initial drug review and approval, while the Drug Safety and Risk Management Advisory Committee provides expert input on safety concerns www.fda.gov/about-fda/center-drug-evaluation-and-research-cder/office-new-drugs-ond-annual-reports; March 14, 2026).

The FDA and EMA both oversee the approval of new medicines. Under its standard pathway, the FDA typically takes about 10 months to review an NDA, while priority review shortens this to around 6 months. Expedited programs like Fast Track, Breakthrough Therapy, and Accelerated Approval can further reduce development and review times. The EMA’s standard centralized procedure takes about 210 days for scientific review, with additional time for applicant responses and European Commission decision-making, often totaling 10–12 months. EMA’s accelerated assessment can reduce review time to 150 days [17, 18].

In terms of identifying safety and efficacy signals, both agencies have robust systems. The FDA uses a structured Benefit-Risk Framework and tends to be more receptive to single-arm trials in areas like oncology. It requires regular safety updates, including expedited reports for serious adverse events and post-marketing surveillance through Risk Evaluation and Mitigation Strategies (REMS). The EMA employs a multi-layered benefit-risk assessment, often requiring comparative data and emphasizing long-term safety. It mandates Risk Management Plans (RMPs), periodic safety updates, and additional monitoring for certain products. Both agencies have strong track records.

The FDA process for approving cancer drugs is different from its process for approving other drugs, primarily due to the frequent use of expedited pathways such as Accelerated Approval, Priority Review, Fast Track, and Breakthrough Therapy designations [19]. For most drugs, the FDA requires substantial evidence of safety and efficacy from at least two adequate and well-controlled clinical trials, with approval typically based on clinical endpoints that directly measure patient benefit (e.g., survival, symptom improvement) [20]. By contrast, cancer drugs are often approved based on surrogate endpoints (e.g., tumor response rate, progression-free survival) that are “reasonably likely” to predict clinical benefit, especially under the Accelerated Approval pathway. This allows for earlier market access but requires post-marketing confirmatory trials to verify actual clinical benefit [21].

### Comparative and complementary value of pharmacovigilance tools

VigiBase, the FDA (via FAERS), and the EMA (via EudraVigilance) are major pharmacovigilance systems used to identify drug toxicity after approval, but they differ in scope, data sources, and regulatory integration.

VigiBase offers international breadth for detecting rare, serious, and geographically diverse adverse events, including those related to cancer drugs that may not be captured in national or regional systems. It uses disproportionality analysis (e.g., Information Component) to identify safety signals and has demonstrated the ability to detect both known and novel toxicities, including cardiac and non-cardiac events associated with cancer drugs [22]. Its global coverage can reveal signals earlier or in populations underrepresented in US or EU data.

FDA’s FAERS is a national system focused on US patients and providers. It is tightly integrated into the FDA’s regulatory process, supporting post-marketing surveillance, label changes, and risk mitigation strategies. The FDA also uses active surveillance tools (e.g., Sentinel Initiative) to complement spontaneous reporting, enabling more robust detection of safety signals in real-world US populations [23].

EMA’s EudraVigilance collects reports from EU member states. It is closely linked to the EMA’s regulatory framework, including post-marketing requirements and risk management plans for cancer drugs [24]. EudraVigilance supports the Pharmacovigilance Risk Assessment Committee in ongoing safety evaluation and regulatory decision-making.

Comparative studies show overlap in safety signals detected by VigiBase, FAERS, and EudraVigilance, with VigiBase often identifying a broader range of signals due to its global scope, while FAERS and EudraVigilance are more closely tied to regulatory actions in their respective regions [25]. Combining data from all three systems increases statistical power and diversity of detected adverse events [26]. The complexity of combining databases will be discussed further in part 3 of this series.

FAERS collects individual case safety reports (ICSRs) from healthcare professionals, manufacturers, and consumers [27]. Reports can be submitted via the FDA’s MedWatch portal, postal mail, fax, or directly to manufacturers, who are required to forward them to the FDA. Each report includes patient demographics, medical history, concomitant medications, detailed descriptions of the AEs and outcomes. Events are coded using the Medical Dictionary for Regulatory Activities (MedDRA), allowing for standardized signal detection and analysis [28].

While the 2007 FDA Amendments Act (FDAAA), which expanded FDA’s postmarketing safety authorities and introduced tools like the Sentinel Initiative (see below), did not change the timing and frequency of safety-related actions for new drugs, there was a reduced time for drugs designated for fast track, breakthrough therapy designation, and accelerated approval drugs. The overall lack of significant improvement in time to first safety action that required up to 3 years indicated that a passive surveillance system would not be sufficient for the development and clinical use of cancer therapeutics [29].

## Evaluation and reporting

Each year, millions of ICSRs are submitted globally for cancer drugs and other pharmaceuticals. These reports document suspected ADRs experienced by individual patients and are collected from healthcare professionals, patients, and pharmaceutical companies. Regulatory databases house tens of millions of ICSRs, with cancer drugs representing a significant portion due to their complex safety profiles and widespread use [30].

Once received, ICSRs undergo a rigorous multi-step processing workflow. First, the report is validated to ensure it contains the four essential elements: an identifiable patient, an identifiable reporter, a suspected drug, and an adverse event. The case is then triaged based on seriousness and urgency-fatal or life-threatening events are flagged for expedited reporting, often within 7 to 15 days depending on regulatory guidelines. After triage, pharmacovigilance professionals enter the case into a safety database, coding adverse events using the Medical Dictionary for Regulatory Activities (MedDRA) and drugs using standardized dictionaries. A medical reviewer assesses causality and expectedness, and a narrative is written to summarize the clinical course. The case is then quality-checked and submitted electronically to regulatory authorities using standardized formats like ICH E2B (R3) (Fig. 1).

Regulatory agencies, specifically the FDA previously reported adverse events quarterly. These reports include serious and unexpected adverse events, including cardiotoxicity, and are submitted by sponsors, healthcare providers, and manufacturers. Starting April 2026, all IND safety reports must be submitted electronically to FAERS, streamlining the process for tracking and analyzing cardiotoxic events.

In August 2025, the FDA decided to release adverse-event data from FAERS *daily* instead of quarterly. This step may increase transparency and allow external stakeholders to identify potential safety signals more quickly. It does not specifically address fundamental limitations of FAERS, such as lack of denominator data, reporting bias, duplicate or incomplete reports, and inability to establish causality [31]. Some have cautioned that daily releases could lead to misinterpretation, misinformation, and even manipulation of data for market or legal purposes. Furthermore, the FDA may lack sufficient resources to review and contextualize data before publication, especially after recent staffing reductions. There is a consensus in the clinical community that rather than focusing on frequency of FAERS data releases, the FDA should invest in modernizing its *active* surveillance system. For example, the Sentinel System could incorporate richer clinical data and advanced analytics. This would enable more precise detection of safety signals, including severity, dose-response, and drug–drug interactions, and improve regulatory decision-making [31]. Within Sentinel, the Active Risk Identification and Analysis (ARIA) system supports safety-signal evaluation by querying routinely collected health care data. However, ARIA is often considered insufficient for thorough evaluations because it relies heavily on claims data and lacks linkage to richer clinical sources such as detailed electronic health records, imaging, and laboratory results.

The recent action of releasing FAERS data daily is also not likely to significantly improve the identification of cardiotoxicity or its timely communication to prescribing physicians. FAERS remains a passive system with inherent limitations, and daily updates do not enhance the FDA’s ability to detect or validate complex adverse events like cardiotoxicity, which often require detailed clinical context and longitudinal data. For oncologists and the cardiology community, actionable insights depend on robust active surveillance systems and real-world evidence, not raw, unverified reports.

### Finalized case report actions

The recipients of these finalized reports include internal divisions within the FDA such as CDER and the Center for Biologics Evaluation and Research (CBER). Additionally, data from FAERS is publicly accessible and used by academic researchers, industry analysts, and international regulatory bodies for pharmacovigilance and risk assessment (www.fda.gov/drugs/drug-approvals-and-databases/fda-adverse-event-reporting-system-faers-database; accessed March 14, 2026). The responsibility for publishing new summaries of cardiotoxicity events in the public domain lies with multiple entities. The FDA itself publishes safety communications and hosts public workshops, such as those focused on cardiovascular toxicity in oncology trials. Journals like *Cardiovascular Toxicology* also play a key role by publishing peer-reviewed studies that analyze cardiotoxicity mechanisms and trends. Additionally, the CDC’s Morbidity and Mortality Weekly Report (MMWR) may publish relevant findings when cardiotoxicity intersects with broader public health concerns. Once a signal for cardiotoxicity is identified, the FDA disseminates this information to physicians and the public via several mechanisms, including Drug Safety Communications, updates to drug labeling (such as black box warnings), press releases, and targeted outreach through professional channels and social media. Drug Safety Communications are the main vehicle for broad dissemination. These communications describe the safety issue, provide clinical recommendations, and summarize the data reviewed. They are distributed via the FDA website, email Listservs, and direct notifications to healthcare providers. For urgent or high-risk events, the FDA may issue immediate press releases or media briefings [32].

### Public reporting on safety

The FAERS Public Dashboard (www.fda.gov/drugs/fdas-adverse-event-reporting-system-faers/fda-adverse-event-reporting-system-faers-public-dashboard; accessed March 14, 2026) is an interactive web-based tool that allows users to search and visualize adverse event reports submitted to the FDA by healthcare providers, consumers, and manufacturers. It includes data on drugs and biologics, and users can filter by drug name, reaction type, patient demographics, and outcomes. However, the dashboard does not confirm causality. For more advanced access, FAERS data is also available through Data Files: These downloadable files (in XML, ASCII, and JSON formats) contain structured data on adverse events, including tables for patient demographics (DEMO), drug details (DRUG), reactions (REAC), and outcomes (OUTC); OpenFDA API: This allows developers and researchers to programmatically query FAERS data in real time. It supports filtering by drug name, reaction, report date, and more, making it suitable for custom dashboards or automated surveillance systems (www.open.fda.gov/data/faers/; accessed March 14, 2026).

### Translation to patient care

Implementation involves continuous data intake, coding, and aggregation. The FDA uses advanced data mining algorithms, such as proportional reporting ratio (PRR), reporting odds ratio (ROR), and empirical Bayes geometric mean (EBGM) to identify statistical associations between drugs and adverse events, which may indicate safety signals [33].Validation of FAERS for cancer drug safety relies on the completeness and quality of submitted reports, as well as corroboration with other data sources (e.g., Sentinel System, published literature) when a signal is detected. Regulatory actions, such as label changes or REMS, are taken when detailed assessment of FAERS data suggests a significant safety concern [34].

Inclusion of cardiovascular safety endpoints is NOT a formal requirement of the FDA or EMA for all oncology drug approvals. The FDA and EMA may request cardiovascular safety studies if there is a plausible signal or mechanistic concern, but routine prospective cardiovascular safety assessment is not mandated for all cancer therapies [35–37].

## Grading systems for drug toxicity

The FDA and the EMA both rely on the National Cancer Institute’s Common Terminology Criteria for Adverse Events (CTCAE) as the standard grading system to classify and report toxicity in cancer clinical trials. The CTCAE provides a comprehensive library of approximately 800 discrete adverse events (AEs), each graded on a 5-point ordinal scale (Grade 1–5) anchored to specific clinical criteria, ranging from mild (Grade 1) to death related to AE (Grade 5) [38]. This system covers laboratory abnormalities, objective clinical findings, and subjective symptoms (Table 2).

**Table 2:** Grading Scale of Drug Toxicity, General Signs and Symptoms, and Clinical Response

Grade	Severity	Signs/Symptoms	Clinical Guidance
Grade 1	Mild	Asymptomatic or mild symptoms; abnormal labs without clinical impact	Continue treatment, monitor. No dose adjustment needed.
Grade 2	Moderate	Symptoms requiring minimal, local, or noninvasive intervention; may limit instrumental ADLs (e.g., shopping, cooking)	Consider holding treatment temporarily; resume at same or reduced dose if symptoms resolve.
Grade 3	Severe	Medically significant but not immediately life-threatening; hospitalization may be required; limits self-care ADLs (e.g., bathing, dressing)	Hold treatment, resume at reduced dose or switch therapy after resolution. Consider specialist consultation.
Grade 4	Life-threatening	Urgent intervention required; ICU-level care may be needed	Discontinue drug permanently or switch to alternative therapy. Manage acute toxicity aggressively.
Grade 5	Death	Death related to adverse event	Not applicable; report and investigate.

ADL: activity of daily living, ICU: intensive care unit

For symptomatic AEs, the CTCAE has historically relied on investigator reporting, but evidence shows that clinicians may underreport or misclassify these events. To address this, the FDA and EMA have endorsed the use of patient-reported outcomes (PROs), specifically the Patient-Reported Outcomes version of the CTCAE (PRO-CTCAE), which allows direct patient input on the frequency, severity, and the interference to daily activities and the quality of life of symptoms [39]. The PRO-CTCAE includes plain language items and standardized recall periods, improving the reliability and completeness of toxicity data.

The CTCAE and PRO-CTCAE frameworks are integrated into regulatory submissions and clinical trial protocols, ensuring harmonized toxicity reporting across global oncology studies. The EMA also supports the use of PROs and has linked the European Organization for Research and Treatment of Cancer (EORTC) Item Library to CTCAE for standardized symptom classification [40].

## Underestimating the true incidence of cardiotoxicity

There are several tools in place to detect cardiotoxicity (Table 3). The topic will be discussed in further detail.

**Table 3:** Tools for Detecting Cancer Drug Cardiotoxicity

Method/Tool	Description	Responsible Party
FDA Adverse Event Reporting System (FAERS)	Voluntary reporting of adverse events and medication errors by providers, patients, and industry; used for signal detection of rare or unexpected events.	FDA, Manufacturers, Providers
Sentinel System	Active surveillance using large-scale electronic health data (claims, EHRs) to detect safety signals in real-world populations.	FDA
Post-marketing Requirements (PMRs)	Legally mandated studies (e.g., new clinical trials, registries, observational studies) to address specific safety or efficacy questions.	FDA, Manufacturers
Post-marketing Commitments (PMCs)	Voluntary or agreed-upon studies are not legally required but conducted to further characterize safety/efficacy.	Manufacturers, FDA
Risk Evaluation and Mitigation Strategies (REMS)	Special programs (e.g., restricted distribution, education) required for drugs with serious safety concerns.	FDA, Manufacturers
ClinicalTrials.gov Registration	Public registration and results reporting of post-marketing studies required by law for many studies.	Manufacturers
MedWatch	FDA’s portal for healthcare professionals and consumers to report adverse events directly.	FDA, Providers, Patients
Labeling Changes/Boxed Warnings	Regulatory actions based on new safety data, including label updates and boxed warnings.	FDA

Food and Drug Administration: FDA

Cardiotoxicity associated with cancer therapies is frequently under-recognized during NDA approval clinical trials, leading to an underestimation of cardiovascular risks that only become apparent through post-market surveillance. Trials often exclude patients with pre-existing cardiovascular conditions and may lack long-term follow-up or sensitive cardiac endpoints, resulting in a skewed safety profile [41, 42]. For example, immune checkpoint inhibitors (ICIs) and tyrosine kinase inhibitors (TKI’s) initially appeared safe in pivotal trials but were later linked to significant cardiac events such as myocarditis and left ventricular dysfunction at much higher rates in real-world settings [43]. This discrepancy underscores the need for clinicians to maintain a high index of suspicion for cardiotoxicity, even when drugs are FDA-approved, and to implement proactive cardiovascular monitoring and risk stratification throughout cancer treatment. Several factors contribute to the recognized underestimation in detection and cardiotoxicity.

First, fast track approval processes often rely on smaller, shorter-duration clinical trials with highly selected patient populations, which may not capture the full spectrum or delayed onset of cardiotoxic events seen in broader, real-world populations. Preclinical models and early-phase trials may not fully predict the range or incidence of cardiovascular toxicities, particularly for novel targeted agents and immunotherapies, which can manifest with diverse and sometimes later-onset cardiac complications [44].

Second, patients with advanced cancer or prior treatment failure frequently have multiple comorbidities and may be receiving combination regimens, making attribution of cardiac events to a specific agent challenging. Cardiotoxicity can be masked by overlapping symptoms of cancer progression or other organ dysfunction, and subclinical injury may go undetected without systematic biomarker or imaging surveillance [45].

Third, the late effects of cancer therapy, including cardiotoxicity, may only become clinically evident years after treatment, well beyond the typical follow-up period of registration trials [46]. Post-marketing surveillance systems and real-world data are therefore essential to more accurately define the incidence and spectrum of cardiotoxicity, but these data may lag far behind clinical use, especially for rapidly approved agents and common cancers.

Fourth, there are important differences in post-approval monitoring requirements for the most common cancer drug classes approved via expedited FDA pathways. All cancer drugs granted accelerated approval, including TKI’s, monoclonal antibodies (including ICIs), cytotoxic therapies, antibody-drug conjugates, and hormonal therapies are subject to mandatory post-marketing requirements (PMRs), most notably confirmatory clinical trials to verify actual clinical benefit, typically using endpoints such as overall survival or progression-free survival rather than the surrogate endpoints used for initial approval [21].

## The safety lag and its implications

While many newly approved cancer therapies offer substantial benefits in terms of survival and disease control, there remains a troubling gap between regulatory approval and the full characterization of their cardiotoxicity profiles. This delay can compromise patient safety, especially when cardiovascular risks emerge only after widespread clinical use [46].

The average time between the approval of a cancer drug and the recognition of the true incidence and complete range of cardiotoxicity associated with that drug is typically 3 to 5 years. This interval reflects the period required for post-marketing pharmacovigilance data to accumulate and for safety communications, such as black box warnings or label changes to be issued, as demonstrated by analyses of FDA safety communications and real-world event reporting [47].

Initial pivotal trials supporting FDA approval often underreport cardiovascular events due to selective enrollment criteria that exclude patients with pre-existing cardiovascular disease, limited follow-up, and inconsistent event adjudication, resulting in an underestimation of cardiotoxicity at the time of approval [48]. Many cardiotoxic effects, especially those related to newer targeted therapies and immunotherapies, are only fully appreciated after several years of broader clinical use and systematic post-marketing surveillance [49].

Underestimation of cardiotoxicity in NDA clinical trials for cancer drugs can mislead both clinicians and patients by presenting an incomplete picture of cardiovascular risks, leading to a false sense of safety [50]. As a result, clinicians may underemphasize the need for cardiac monitoring, and patients may not receive appropriate screening or preventive care, especially when treated with agents subsequently determined to have delayed or cumulative cardiotoxic effects.

To address this gap, clinicians should actively seek updated data from post-marketing surveillance, real-world registries, and Cardio-Oncology collaborations. These sources often reveal higher incidences of cardiotoxicity than initially reported and provide insights into patient populations more representative of clinical practice. Incorporating advanced imaging techniques, cardiac biomarkers, and guideline-based risk stratification can help tailor surveillance strategies. Staying current with evolving recommendations from expert panels and societies like ASCO and the ESC is essential to ensure that patient discussions and care plans reflect the most accurate and comprehensive understanding of cardiovascular risks associated with cancer therapies [51].

## Cardiotoxicity and labeling changes

Cancer drugs tend to have a higher rate of post-approval safety updates. A cohort study analyzed 382 FDA-approved drugs between 1997 and 2016 and found that those approved via expedited pathways had an average of 0.94 safety-related label changes per year, compared to 0.68 for drugs approved through standard pathways. These expedited drugs also had a 48% higher rate of changes to the most serious sections of the label, such as Boxed Warnings and Contraindications [52].

A real-world analysis of FAERS data focusing on CDK4/6 inhibitors such as palbociclib, ribociclib, and abemaciclib revealed 2,065 cardiac adverse events out of 69,139 total reports. These events included hypertension, heart failure, QT prolongation, and cardiomyopathy. The median time to onset was 69 days, but the range extended from days- to- years. Ribociclib showed a strong association with QT prolongation, with a proportional reporting ratio (PRR) of 8.43 and a reporting odds ratio (ROR) of 8.65 [53].

The evidence suggests that cancer drugs are likely to undergo multiple safety-related updates in the first 10 years post-approval. Based on current data, it is reasonable to expect one to three cardiotoxicity-related labeling changes per drug during this period, especially for agents with known or emerging cardiac risks.

## Drug approvals, rejections, and withdrawals

The most common reasons for non-approval of cancer drugs differ by both geographic region and cancer type, but several consistent themes emerge across regulatory agencies [54]. In the U.S., FDA non-approval is most frequently due to insufficient evidence of clinical benefit, particularly when pivotal trials rely on surrogate endpoints (such as progression-free survival or objective response rate) that do not convincingly translate to improvements in overall survival or quality of life. Safety concerns, including significant adverse events, and issues with trial design (such as lack of randomization or inadequate control arms) are also important factors [55].

In Europe (EMA and NICE [National Institute for Health and Care Excellence] in England), cost-effectiveness is a reason for non-approval, in addition to the clinical benefit and safety concerns. Briefly, NICE is best known for its role in evaluating the clinical and cost-effectiveness of medicines, treatments, and procedures. Its recommendations help the National Health Service (NHS) decide which treatments should be made available to patients. NICE uses rigorous evidence-based assessments, including health technology assessments (HTAs), to determine whether a drug or intervention offers good value for money and improves patient outcomes [56].

Over the past 10 years (2015–2025), no cancer drugs were denied approval by the FDA or EMA solely due to early cardiotoxicity signals; regulatory agencies have generally required additional cardiac safety data or imposed post marketing requirements rather than outright rejection for cardiotoxicity alone [57, 58].

This approach is supported by the recognition that preclinical models of cardiotoxicity, as highlighted by the AHA and summarized below, have important limitations in predicting human risk, especially given the complexity of cancer therapy-associated cardiovascular toxicity and the need to balance antitumor efficacy with cardiac safety [59]. Regulatory decisions thus reflect a pragmatic balance: the potential for substantial clinical benefit in oncology often outweighs early, non-definitive cardiotoxicity signals, provided that robust post-marketing surveillance and risk mitigation strategies are in place [60]. The agencies have not fundamentally changed preclinical or NDA-enabling study requirements because current models do not reliably predict clinical cardiotoxicity, and the evolving landscape of cancer therapeutics demands ongoing real-world safety monitoring and risk stratification [61].

However, several drugs were withdrawn from the market after approval because of cardiotoxicity, and many were approved with sentinel programs (e.g., black box warnings, Risk Evaluation and Mitigation Strategies [REMS], or mandatory cardiac monitoring) due to cardiotoxicity concerns (Table 4).

**Table 4:** Cancer Drugs Withdrawn from the Market Post-Approval due the Cardiotoxicity

Drug Name	Regulatory Outcome	Cardiotoxicity Concern	Cancer Type(s)	Region(s)
Ponatinib	Withdrawn (some indications)	Arterial thrombosis, HF	Leukemia	US, EU
Vandetanib	Withdrawn (some regions)	QT prolongation, SCD	Thyroid	US, EU
Doxorubicin	Approved w/ sentinel program	Cardiomyopathy, HF	Multiple	US, EU
Epirubicin	Approved w/ sentinel program	Cardiomyopathy, HF	Multiple	US, EU
Trastuzumab	Approved w/ sentinel program	LV dysfunction, HF	Breast, others	US, EU
Pertuzumab	Approved w/ sentinel program	LV dysfunction, HF	Breast	US, EU
Lapatinib	Approved w/ sentinel program	QT prolongation, HF	Breast	US, EU
Sunitinib	Approved w/ sentinel program	HF, hypertension, QT prolong.	Renal, others	US, EU
Sorafenib	Approved w/ sentinel program	HF, hypertension, QT prolong.	Renal, liver	US, EU
Pazopanib	Approved w/ sentinel program	QT prolongation, HF	Renal, soft tissue	US, EU
Nilotinib	Approved w/ sentinel program	QT prolongation, vascular	Leukemia	US, EU
Dasatinib	Approved w/ sentinel program	Pulmonary hypertension	Leukemia	US, EU
Ibrutinib	Approved w/ sentinel program	Atrial fibrillation	Lymphoma, leukemia	US, EU

HF: Heart Failure, SCD: Sudden Cardiac Death, LV: left ventricular, US: United States, EU: European Union

Drugs withdrawn post-approval due to cardiotoxicity includes ponatinib (for certain indications, due to arterial thrombotic events and heart failure) and vandetanib (withdrawn in some regions due to QT prolongation and sudden cardiac death risk). Drugs approved with sentinel programs for cardiotoxicity include anthracyclines (doxorubicin, epirubicin), HER2-targeted agents (trastuzumab, pertuzumab, lapatinib), VEGF/PDGF inhibitors (sunitinib, sorafenib, pazopanib, vandetanib), BCR-ABL inhibitors (nilotinib, dasatinib, ponatinib), and BTK inhibitors (ibrutinib), all of which carry boxed warnings or require cardiac monitoring due to risks such as heart failure, arrhythmias, QT prolongation, and vascular events [62–65].

Drugs approved with sentinel programs for cardiotoxicity include anthracyclines (doxorubicin, epirubicin), HER2-targeted agents (trastuzumab, pertuzumab, lapatinib), VEGF/PDGF inhibitors (sunitinib, sorafenib, pazopanib, vandetanib), BCR-ABL inhibitors (nilotinib, dasatinib, ponatinib), and BTK inhibitors (ibrutinib), all of which carry boxed warnings or require cardiac monitoring due to risks such as heart failure, arrhythmias, QT prolongation, and vascular events.

## Cardiotoxicity of cancer drugs: Clinician detection

Cardiotoxicity has become more common with contemporary cancer treatments compared to the past. This increase is largely due to the introduction of new chemotherapy and immunotherapy agents that, while effective in treating cancer, often come with severe side effects, including cardiotoxicity [66]. Clinician and patient awareness coupled with monitoring and surveillance strategies also contribute to higher incidence [67]. It is important for clinicians to be cognizant of the grades or severity of drug toxicity when considering benefits, risks, and shared decision making (see prior section on Toxicity Grading System).

## Strengths and limitations of post-approval safety assessment

The strengths of post-approval processes for detecting cardiotoxicity in cancer drugs include the ability to capture real-world cardiovascular events across diverse patient populations, including those with comorbidities and prior cardiac history who are often excluded from early-phase trials. Large multicenter registries and post-marketing surveillance can identify rare, delayed, or cumulative toxicities, and facilitate ongoing risk stratification and refinement of monitoring protocols [68]. The ASCO recommends serial imaging and biomarker surveillance (troponins, natriuretic peptides) for early detection of cardiac dysfunction in patients receiving cardiotoxic therapies, which can be implemented in routine clinical practice post-approval. Advances in cardiovascular imaging and molecular biomarkers further enhance sensitivity for subclinical injury and allow for more personalized monitoring [69].

Limitations include under-reporting and inconsistent adjudication of cardiovascular events, heterogeneity in diagnostic criteria, and lack of standardized definitions across oncology and cardiology domains. The CTCAE used in oncology trials may not align with cardiovascular standards, leading to misclassification or missed events [69]. Surveillance is often resource-intensive, and imaging or biomarker strategies may not be uniformly applied, especially outside academic centers [70]. Additionally, post-approval detection is inherently reactive, identifying toxicity only after patient exposure, and may miss subtle or long-term effects that were signaled in preclinical or early-phase studies but not systematically followed [71].

## The evolution of regulatory assessment of cancer drugs

Both the FDA and the EMA have taken steps over the past five years to better determine cardiotoxicity early in cancer drug development, but these changes are still evolving and not yet fully standardized.

The FDA, through its Oncology Center of Excellence, has launched initiatives such as *Project Optimus*, which aims to reform early-phase oncology trials by requiring more robust dose optimization studies that include comprehensive toxicity assessments, including cardiotoxicity. International regulatory agencies, including the EMA, are monitoring and considering similar reforms, especially through collaborative programs like Project Orbis, which facilitate coordinated review and alignment of safety standards [72].

Despite these policy shifts, systematic reviews of regulatory practice indicate that while benefit-risk assessments have diversified, explicit integration of early cardiotoxicity data into approval decisions remains inconsistent. The standard of evidence for cardiovascular safety is improving, but patient experience and real-world data are still rarely incorporated into regulatory benefit-risk assessments [57]. Furthermore, post-marketing safety communications regarding cardiovascular events remain common, with cardiac safety warnings issued for over half of novel cancer therapies approved by the FDA, highlighting the ongoing need for earlier and more rigorous cardiotoxicity evaluation [73] (Table 5).

**Table 5:** Alternative Drug Approval and Safety Surveillance Models

Model	Key Features	Strengths	Challenges
European Medicines Agency (EMA)	Centralized EU drug approval, strong post-market surveillance	High scientific rigor, transparency	Slower approval timelines, limited U.S. jurisdiction
Private Certification Bodies	Independent safety/efficacy review, industry-funded	Potentially faster, market-driven	Risk of bias, lack of unified standards
Blockchain Pharmacovigilance	Decentralized tracking of adverse events and manufacturing data	Transparency, tamper-resistance	Technical complexity, adoption barriers
Decentralized Clinical Trials	Remote participation, real-world data collection	Inclusive, cost-effective	Data integrity, regulatory oversight gaps
Academic/Nonprofit Oversight	Peer-reviewed trials, independent monitoring boards	Scientific credibility, public trust	Limited scalability, funding constraints
Global Surveillance Networks	WHO’s VigiBase and similar systems for adverse event reporting	International collaboration, broad data pool	Data harmonization, limited enforcement power

U.S.: United States, WHO: World Health Organization

## Conclusions

The current regulatory frameworks for oncology drug development, while effective in accelerating access to life-saving therapies remain insufficient for early detection and characterization of cardiotoxicity. Reliance on surrogate endpoints, exclusion of high-risk patients with cardiovascular disease, and limited integration of cardiovascular safety measures in expedited pathways contribute to underestimation of cardiac risk. Post-marketing surveillance systems such as FAERS, EudraVigilance, and VigiBase provide essential safety monitoring but are inherently passive by design, reactive and constrained by underreporting and data heterogeneity. These gaps underscore the urgent need for proactive, system-level strategies that harmonize regulatory science with real-world cardiovascular risk to optimize therapeutic benefit-risk balance.

Regulatory modernization is equally critical; initiatives such as the FDA’s Project Optimus, an important upstream activity in drug development, should be expanded to mandate dose optimization and comprehensive toxicity profiling, including cardiotoxicity, before market entry. Beyond trial data, real-world evidence should play a central role in active surveillance systems, leveraging electronic health records, registries, and advanced analytics to enable timely detection of cardiovascular signals. Global harmonization of safety standards between agencies such as the FDA and EMA will reduce variability and improve patient outcomes, while collaborative models involving regulators, academic centers, and industry can accelerate the development of predictive tools and shared data platforms for cardiotoxicity risk stratification.

Innovative approaches to the regulatory process of cancer therapeutics with a goal of increasing and accelerating the detection of cardiotoxicity are the topic of the third paper in this series.
